# Maternal Methyl Donor Nutrients Modulate Developmental Genes in Mammary Tumors

**DOI:** 10.3390/biology15080645

**Published:** 2026-04-19

**Authors:** Lawrence Mabasa, Anri Kotze, Rabia Johnson, Pritika Ramharack, Sylvester I. Omoruyi, Kwazikwakhe B. Gabuza, Jyoti Sharma, Tarryn Willmer

**Affiliations:** 1 Biomedical Research and Innovation Platform, South African Medical Research Council, Tygerberg, Cape Town 7505, South Africa; anri.kotze@mrc.ac.za (A.K.); rabia.johnson@mrc.ac.za (R.J.); pritika.ramharack@mrc.ac.za (P.R.); kwazi.gabuza@mrc.ac.za (K.B.G.); jyoti.sharma@mrc.ac.za (J.S.); tarryn.willmer@mrc.ac.za (T.W.); 2 Department of Medical Physiology, Stellenbosch University, Tygerberg, Cape Town 7507, South Africa; 3 Department of Anatomical Sciences, School of Biomedical Sciences, Faculty of Health Sciences, University of the Witwatersrand, Parktown, Johannesburg 2193, South Africa; sylvester.omoruyi@wits.ac.za; 4 Division of Cell Biology, Department of Human Biology, Faculty of Health Sciences, University of Cape Town, Cape Town 7925, South Africa

**Keywords:** lipotropes, methyl donor nutrients, micronutrients, DOHaD, maternal, mother, child, epigenetics, mammary development, DNA methylation, one-carbon metabolism

## Abstract

Maternal nutrition during critical windows of development plays a pivotal role in shaping long-term disease susceptibility, including cancer risk. This study investigated whether maternal exposure to lipotropes (methyl donor nutrients) during pregnancy and lactation modulates gene expression in 7,12-dimethylbenzanthracene (DMBA)-induced mammary tumors in adult female offspring. Timed-pregnant Sprague-Dawley rats were fed with either a control or lipotrope-supplemented diet, with or without vitamin B6. Female offspring were exposed to DMBA at puberty, and mammary tumors were evaluated histologically and molecularly. DMBA-induced tumors displayed ductal carcinoma in situ-like morphology and significant upregulation of fetal mammary developmental genes (*Tbx2* and *Tbx3*), the tumorigenesis-associated gene *Tp53*, and key epigenetic regulators (*Hdac1*, *Dnmt1*, and *Mthfr*). Estrogen receptor 1 (*Esr1*) mRNA expression also showed a significant increase. Maternal lipotropes supplementation significantly attenuated the expression of these genes in offspring tumors. Collectively, these findings demonstrate that maternal methyl donor nutrition modulates tumor-associated gene expression patterns, potentially by limiting the reactivation of developmental and epigenetic pathways in adulthood. This study highlights maternal nutrition as a modifiable early-life factor with important implications for long-term health programming.

## Simple Summary

Maternal nutrition during early life can have lasting effects on offspring’s health, including breast cancer risk in adulthood. In this study, we show that maternal nutrition during pregnancy influences long-term regulation of mammary gene expression in the offspring, long after exposure. Specifically, maternal methyl donor nutrients affected the reactivation of fetal mammary genes, altered key epigenetic regulators, and disrupted estrogen receptor signaling, all of which are linked to tumor development. These findings suggest that what a mother eats during pregnancy can play an important role in a child’s future health. A healthy maternal diet may influence how breast tissue develops early in life, potentially affecting breast cancer risk later by shaping how certain genes are switched on or off. Thus, promoting optimal maternal nutrition may therefore offer a practical strategy to reduce breast cancer risk across populations.

## 1. Introduction

Breast cancer remains the most diagnosed cancer among women globally, with an estimated 2.3 million new cases reported in 2022 [1]. Despite advances in early detection and treatment, breast cancer incidence continues to rise, underscoring the need for improved prevention strategies, in particular, those that address disease susceptibility earlier in the life course. Breast cancer is a heterogeneous disease driven by a complex interplay of genetic, hormonal, environmental, and lifestyle-related factors, many of which converge on epigenetic regulation of gene expression [2].

The Developmental Origins of Health and Disease (DOHaD) paradigm proposes that environmental exposures during critical periods of development, particularly in utero, can permanently shape disease risk in adulthood [3,4]. Evidence from famine cohorts, including the Dutch Hunger Winter and the Great Chinese Famine, demonstrates that prenatal nutritional perturbations are associated with increased susceptibility to chronic non-communicable diseases later in life [5,6,7,8]. These long-term effects are largely attributed to epigenetic programming, whereby early-life environmental cues modify gene expression without altering the underlying DNA sequence [9].

Among epigenetic mechanisms, DNA methylation is the most extensively studied and is considered a hallmark of cancer development, including breast cancer [10]. Aberrant DNA methylation patterns, characterized by global hypomethylation alongside promoter-specific hypermethylation, contribute to genomic instability, oncogene activation, and tumor suppressor gene silencing [11,12]. Importantly, DNA methylation reactions depend on the availability of methyl groups generated through one-carbon metabolism, a network of interconnected biochemical pathways that rely on adequate levels of methyl donor nutrients, including methionine, folate, choline, and vitamin B_12_.

Experimental studies have demonstrated that maternal supplementation with methyl donor nutrients reduces susceptibility to chemically induced estrogen receptor-positive mammary tumors in female rat offspring [10,13]. Although previous studies, including our own, have established this protective effect, the underlying molecular mechanisms remain poorly understood. The originality of this study lies in elucidating the mechanistic link between early-life maternal nutrition and the long-term regulation of developmental gene networks and epigenetic pathways involved in mammary tumorigenesis.

Emerging evidence suggests that genes essential for fetal mammary gland development may be reactivated or dysregulated during tumorigenesis, thereby linking developmental pathways with cancer biology. In particular, T-box transcription factors 2 and 3 (TBX2 and TBX3), key regulators of embryonic development, have been implicated in breast cancer progression and are subject to epigenetic regulation through DNA methylation [14,15,16,17]. Altered expression of these genes has been associated with estrogen receptor signaling, cell cycle regulation, and tumor aggressiveness, highlighting their potential role as mechanistic mediators of fetal programming effects on breast cancer risk [16,18,19].

In this study, we investigated whether maternal dietary intake of methyl donor nutrients during pregnancy and lactation modulates the expression of fetal mammary developmental genes in DMBA-induced mammary tumors in female rat offspring. We hypothesize that the availability of maternal methyl donor nutrients constrains the tumor-associated reactivation of developmental and epigenetic pathways in adulthood. By specifically examining the dysregulation of fetal mammary developmental genes in carcinogen-induced tumors, this work addresses a critical gap in understanding the developmental origins of breast cancer.

This assessment is particularly important because identifying the molecular pathways through which maternal nutrition influences offspring cancer risk may reveal novel targets for early prevention strategies and strengthen the rationale for nutritional interventions during pregnancy and lactation as a means of reducing breast cancer susceptibility later in life. To model human estrogen receptor-positive breast cancer, 7,12-dimethylbenzanthracene (DMBA), a well-established carcinogen, was used to induce mammary tumors in Sprague-Dawley rats [20,21,22].

## 2. Materials and Methods

### 2.1. Animal Model and Dietary Intervention

All animal experiments were conducted in accordance with institutional and national guidelines for the care and use of laboratory animals and in compliance with approved ethical protocols.

Timed-pregnant Sprague-Dawley rats (10 weeks old) were obtained from the Stellenbosch University Animal Facility (Stellenbosch, Western Cape, South Africa) and housed individually under controlled environmental conditions (temperature 22–25 °C, relative humidity 45–55%, 12 h light/dark cycle). Animals had ad libitum access to food and water throughout the study period.

**Figure 1:**
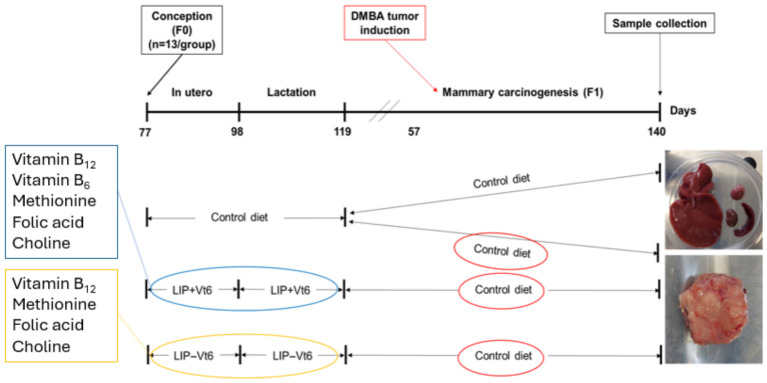
Animal trial. Pregnant Sprague-Dawley rats were assigned to either a control diet, a lipotropes plus vitamin B_6_ (LIP + Vt6)-supplemented diet, or a lipotropes minus vitamin B_6_ (LIP − Vt6)-supplemented diet throughout gestation and lactation. DMBA tumor induction was performed in half of the control offspring and in approximately 7–8 offspring per group in the LIP + Vt6 and LIP − Vt6 groups.

Dams were randomly allocated to one of three experimental diets (Table 1) administered throughout gestation and lactation: (i) a control American Institute of Nutrition (AIN)-93G (New Brunswick, NJ, USA) diet, (ii) a lipotropes-supplemented diet including vitamin B_6_, or (iii) a lipotropes-supplemented diet excluding vitamin B_6_ [Figure 1]. The control AIN-93G diet contained basal levels of methyl donor nutrients, whereas the supplemented diets provided approximately five-fold higher levels of choline, folic acid, vitamin B_6_, and vitamin B_12_, and 1.8-fold higher methionine to prevent potential toxicity, consistent with established protocols [10,13,23].

At parturition, litter size and pup weights were recorded. Where possible, litters were standardized to a maximum of 8 pups per dam to minimize variability in nutritional exposure during lactation. Pups remained with their biological dams until weaning. At postnatal day 21, female offspring were randomly selected, separated from dams, and maintained on the control AIN-93G diet for the remainder of the experimental period.

### 2.2. Mammary Tumor Induction

Mammary carcinogenesis was induced in female offspring at approximately 57 days of age using 7,12-dimethylbenzanthracene (DMBA), a well-established carcinogen that induces estrogen receptor-positive mammary tumors resembling early-stage human breast cancer [20,21,22]. DMBA (Sigma-Aldrich, St. Louis, MO, USA) was dissolved in sunflower oil immediately before administration and delivered by oral gavage at a dose of 45 mg/kg body weight once weekly for three consecutive weeks, following validated protocols (Sheokand et al., 2019) [24]. A subset of age-matched female offspring did not receive DMBA and served as non-cancer controls.

Animals were monitored weekly for general health, body weight changes, and signs of tumor development. Mammary tumors were detected by palpation and measured with Vernier calipers to monitor tumor growth. Humane endpoints, including excessive tumor burden or signs of distress, were applied in accordance with the ethical approvals to minimize animal suffering.

### 2.3. Tissue Collection and Histological Analysis

Animals were euthanized under isoflurane anesthesia followed by exsanguination once experimental or humane endpoint criteria were reached. Mammary glands and tumors were carefully excised, weighed, and processed for downstream analyses. Portions of tissue were fixed in 10% neutral-buffered formalin for histological evaluation, while remaining samples were preserved in RNAlater (Qiagen, Hilden, Germany) and stored at −20 °C for molecular analyses.

Formalin-fixed tissues were paraffin-embedded, sectioned at 4–5 µm thickness, and stained with hematoxylin and eosin (H&E) according to standard histological protocols to assess tissue morphology and confirm tumor histopathology [25].

### 2.4. RNA Extraction and Quantitative Real-Time PCR

Total RNA was extracted from mammary and tumor tissues using the RNeasy Fibrous Tissue Mini Kit (Qiagen, Germany) in accordance with the manufacturer’s instructions. RNA concentration and purity were determined spectrophotometrically, and samples with acceptable purity ratios were used for complementary DNA (cDNA) synthesis. One microgram of total RNA was reverse transcribed using the High-Capacity cDNA Reverse Transcription Kit (Applied Biosystems, Waltham, MA, USA).

Gene expression analysis was performed using quantitative real-time PCR (qRT-PCR) with TaqMan assays on a QuantStudio 7 Flex Real-Time PCR System (Applied Biosystems) (*n* = 7–8 in triplicates per biological sample). Target genes included those involved in fetal mammary development (*Tbx2*, *Tbx3*, *Wnt10b*), epigenetic regulation and one-carbon metabolism (*Hdac1*, *Dnmt1*, *Mthfr*), hormone signaling (*Esr1*), and tumorigenesis-related pathways (*Cdkn1b* and the *p53* tumor suppressor gene). Housekeeping genes, Beta-2-Microglobulin (*B2m*) and Hypoxanthine Phosphoribosyltransferase (*Hprt1*) were used for normalization. Relative gene expression was evaluated using the Relative Standard Curve method, in which standard curves were generated for both target and reference genes using serial dilutions of cDNA. The cycle threshold (Ct) values of samples were interpolated against these curves to determine relative quantities. Expression levels of target genes were then normalized to the endogenous reference gene to account for sample-to-sample variation in cDNA input and amplification efficiency. All reactions were performed in technical replicates, and the resulting normalized values were used for downstream statistical analysis.

### 2.5. Statistical Analysis

All data are presented as mean ± standard error of the mean (SEM). Statistical analyses were conducted using GraphPad Prism software (version 10.6.1). All data were first screened for outliers using Grubbs’ test. Data distribution was then assessed for normality using the Shapiro–Wilk test. For comparisons across multiple groups, we used Welch’s ANOVA, which does not assume equal variances. When a significant overall effect was detected (*p* ≤ 0.05), post hoc multiple comparisons were performed using Dunnett’s T3 test to account for unequal variances between groups. Pearson correlation analysis was performed to assess the relationship between *Esr1* expression with selected developmental, epigenetic, and tumorigenesis-related genes. Statistical significance was set at *p* ≤ 0.05.

## 3. Results

### 3.1. DMBA Induces Estrogen Receptor-Positive Mammary Tumors with Ductal Carcinoma In Situ (DCIS) Morphology

**Figure 2:**
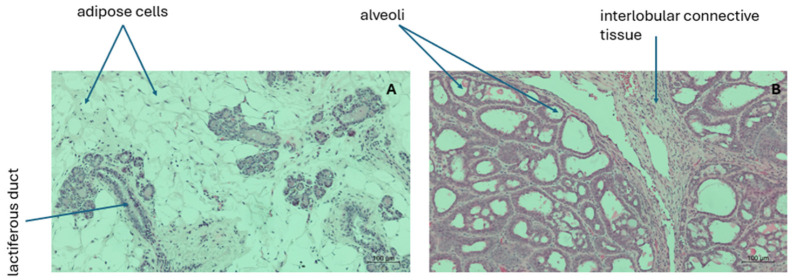
Histological confirmation of DMBA-induced mammary tumorigenesis. Representative hematoxylin and eosin (H&E)-stained (100× magnification sections of mammary tissue from non-cancer controls (**A**) and DMBA-induced mammary tumors (**B**). Non-cancer controls exhibit intact ductal architecture with organized epithelial layers, whereas tumors display DCIS-like features.

Histological examination of hematoxylin and eosin (H&E)-stained tissue sections revealed marked structural differences between non-cancer controls and DMBA-induced mammary tumors (Figure 2). Mammary glands from non-cancer control animals displayed well-defined ductal architecture, characterized by organized epithelial cell layers, intact basement membranes, and preserved stromal architecture. These features are indicative of normal mammary gland morphology. In contrast, mammary tumors from DMBA-treated offspring exhibited pronounced histopathological alterations consistent with ductal carcinoma in situ (DCIS). These changes included ductal enlargement, epithelial hyperplasia with multilayered stratification, increased nuclear-to-cytoplasmic ratio, nuclear pleomorphism, and disruption of normal tissue organization. Collectively, these findings confirm successful induction of estrogen receptor-positive mammary carcinogenesis using the DMBA model and provide a histological foundation for subsequent molecular analyses.

### 3.2. Maternal Methyl Donor Nutrients Exposure Attenuates Reactivation of Fetal Mammary Developmental Genes in Mammary Tumors

**Figure 3:**
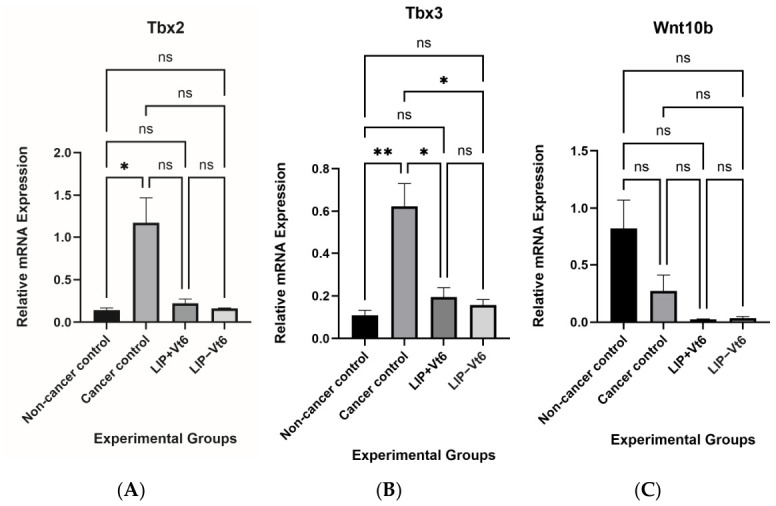
Relative mRNA expression of fetal mammary developmental genes [*Tbx2* (**A**), *Tbx3* (**B**), and *Wnt10b* (**C**)] in mammary tumors and non-cancer mammary tissue. Maternal methyl donor nutrients exposure suppresses tumor-associated reactivation of fetal mammary developmental genes. Results are presented as mean ± SEM using GraphPad Prism 10.6.1 with *n* = 7–8/group, *p* ≤ 0.05 represent statistical significance. Significance is indicated as: * *p* ≤ 0.05; ** *p* ≤ 0.01; ns, not significant. Abbreviations: Tbx2: T-box transcription factor 2; Tbx3: T-box transcription factor 3; LIP + Vt6: lipotropes supplementation including vitamin B_6_; LIP − Vt6: lipotropes supplementation excluding vitamin B_6_.

To determine whether mammary tumorigenesis is associated with the reactivation of developmental pathways, the expression of key fetal mammary developmental genes was assessed. Mammary tumors from the cancer control group demonstrated significantly elevated mRNA expression of *Tbx2* and *Tbx3* compared with non-cancer control mammary tissue (*p* ≤ 0.05; Figure 3). These transcription factors are known regulators of embryonic mammary development and have been implicated in oncogenic reprogramming. Notably, maternal exposure to lipotrope-supplemented diets, both with and without vitamin B_6_, resulted in a significant reduction in tumor *Tbx3* expression, while *Tbx2* tended to be reduced (*p* = 0.073 and 0.052, respectively) as compared with cancer controls (*p* ≤ 0.05).

In contrast, expression of *Wnt10b*, another gene involved in mammary development, was not different between the groups.

Taken together, these findings suggest that DMBA-induced tumorigenesis selectively engages specific developmental regulators rather than globally activating developmental signaling pathways. Further attenuation due to maternal methyl donor nutrients indicates that maternal methyl donor nutrients availability during pregnancy and lactation suppresses tumor-associated reactivation of key fetal mammary developmental genes in adult offspring, highlighting a long-term programming effect of early-life nutrition on tumor gene expression.

### 3.3. Maternal Lipotropes Modulate Epigenetic Regulation of Genes Involved in Mammary Tumor Growth

**Figure 4:**
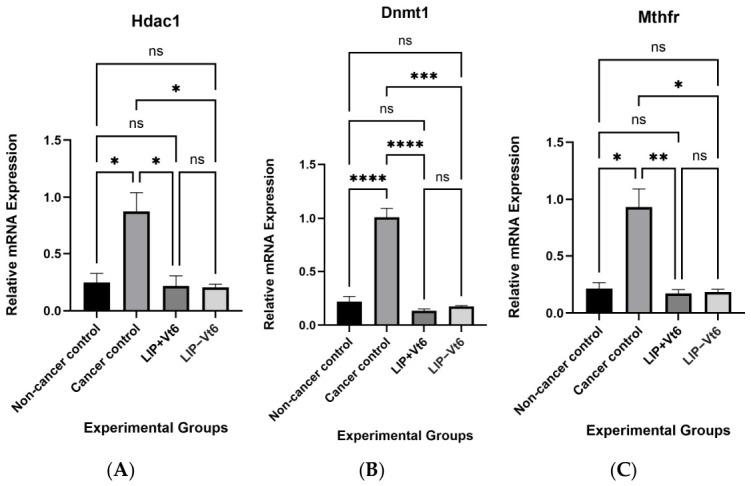
Relative mRNA expression of epigenetic and one-carbon metabolism-related genes [*Hdac1* (**A**), *Dnmt1* (**B**), and *Mthfr* (**C**)] in mammary tumors. Maternal lipotropes supplementation modulates epigenetic regulation of genes involved in tumor growth. Results are presented as mean ± SEM using GraphPad Prism 10.6.1 with *n* = 7–8/group, *p* ≤ 0.05 represent statistical significance. Significance is indicated as follows: * *p* ≤ 0.05; ** *p* ≤ 0.01; *** *p* ≤ 0.001; **** *p* ≤ 0.0001. ns, not significant. Abbreviations: *Hdac1*: histone deacetylase 1; *Dnmt1*: DNA methyltransferase 1; *Mthfr*: methylenetetrahydrofolate reductase; LIP + Vt6: lipotropes supplementation including vitamin B_6_; LIP − Vt6: lipotropes supplementation excluding vitamin B_6_.

Given the dependence of epigenetic processes on one-carbon metabolism, genes involved in chromatin modification and DNA methylation were examined to assess potential mechanisms underlying the observed developmental gene modulation. Mammary tumors from the cancer control group exhibited significantly increased expression of *Hdac1* and *Dnmt1* compared with non-cancer controls (*p* ≤ 0.05; Figure 4), reflecting enhanced chromatin remodeling and maintenance of DNA methylation patterns during tumorigenesis. Additionally, expression of *Mthfr*, a key enzyme regulating methyl group availability within the folate cycle, was significantly elevated in cancer control tumors (*p* ≤ 0.05), suggesting increased metabolic demand for methyl donor nutrients in tumor tissue.

Importantly, maternal lipotropes supplementation significantly attenuated the expression of *Hdac1*, *Dnmt1*, and *Mthfr* in mammary tumors compared with cancer controls (*p* ≤ 0.05). These findings indicate that intrauterine exposure to methyl donor nutrients influences the long-term regulation of epigenetic machinery and one-carbon metabolism in mammary tissue, potentially limiting tumor-associated epigenetic reprogramming in adulthood.

### 3.4. Altered Expression of Tumorigenesis-Related Genes

**Figure 5:**
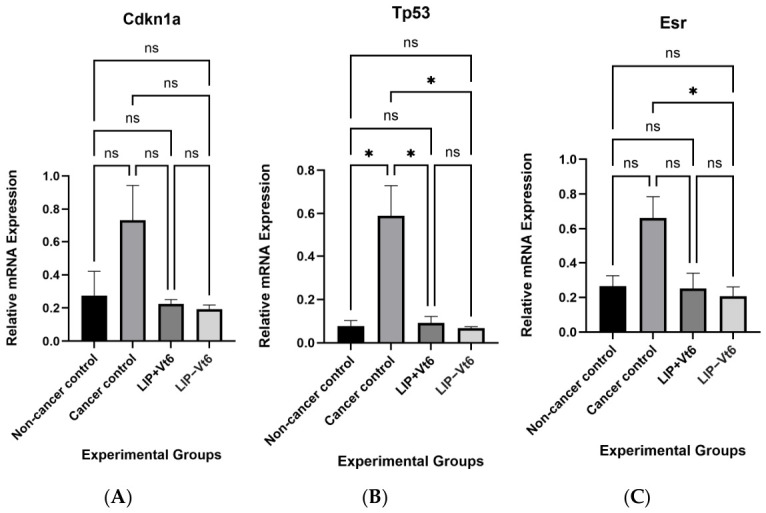
Relative mRNA expression of tumorigenesis-related genes (*Cdkn1a* (**A**), *Tp53* (**B**), and *Esr1* (**C**)) in mammary tumors. Maternal lipotropic nutrients attenuated tumorigenesis-related gene expression. Results are presented as mean ± SEM using GraphPad Prism 10.6.1 with *n* = 7–8/group, *p* ≤ 0.05 represent statistical significance. Significance is indicated as: * *p* ≤ 0.05; ns, not significant. Abbreviations: *Cdkn1a*: cyclin-dependent kinase inhibitor 1a, also known as tumor protein 21, *p21*; *Tp53*: tumor protein 53; *Esr1*: estrogen receptor 1; LIP + Vt6: lipotropes supplementation including vitamin B_6_; LIP − Vt6: lipotropes supplementation excluding vitamin B_6_.

To further evaluate the impact of maternal micronutrient supplementation on tumor biology, the expression of genes involved in cell cycle regulation and tumor suppression was assessed. Mammary tumors from the cancer control group exhibited no significant differences in the expression of *Cdkn1a* (*p21*) expression compared with non-cancer controls (Figure 5). Interestingly, *Tp53* (*p53*) expression was significantly higher in cancer controls relative to non-cancer controls (*p* ≤ 0.05). Similarly, *Esr1* expression tended to be elevated in cancer controls, but this difference was not statistically significant (*p* = 0.09).

Notably, for *Tp53*, both supplemented groups presented with a significant reduction in *Tp53* expression as compared with the cancer control (*p* ≤ 0.05). Similarly, *Esr1* expression was significantly reduced in tumors from the lipotropes minus vitamin B_6_ group compared with cancer controls (*p* ≤ 0.05), whereas tumors from the lipotropes plus vitamin B_6_ group showed a tendency to be lower (*p* = 0.1).

Collectively, these findings indicate that maternal micronutrient supplementation, particularly lipotropes with or without vitamin B_6_, may influence tumor biology by modulating the expression of key genes associated with tumor suppression and estrogen signaling. The observed reductions in *Tp53* and *Esr1* expression in supplemented groups suggest a potential alteration in tumor regulatory pathways, highlighting the importance of maternal diet in shaping offspring tumor gene expression profiles and underscoring the need for further mechanistic investigation.

### 3.5. Maternal Methyl Donor Nutrients Modulation of Esr1-Linked Developmental and Tumorigenic Pathways

Estrogen receptor 1 (*Esr1*) was selected as the reference gene for correlation analyses because of its central role in estrogen signaling, including regulation of direct transcriptional targets and genes modulated through pathway crosstalk (Table 2). Assessing correlations between *Esr1* and developmental regulators (*Tbx2*, *Tbx3*, *Wnt10b*), cell cycle and tumor suppressor genes (*Cdkn1a*, *Tp53*), and epigenetic modifiers (*Dnmt1*, *Hdac1*, *Mthfr*) enables evaluation of coordinated transcriptional networks. These relationships may shift during tumorigenesis and in response to maternal nutritional modulation, providing insight into how prenatal methyl donor nutrients exposure influences estrogen receptor-driven molecular pathways.

In the non-cancer control group, *Esr1* expression showed a positive correlation with *Tbx2* (r = 0.68) that demonstrated a statistical tendency (*p* = 0.09), and a strong positive correlation with *Tbx3* (r = 0.89) that was statistically significant (*p* ≤ 0.05). In the cancer control group, *Esr1* expression was significantly positively correlated with *Tbx2* (r = 0.74; *p* ≤ 0.05), indicating a significant association under tumor conditions. Under lipotropes supplementation excluding vitamin B_6_ (LIP − Vt6), *Esr1* exhibited a positive correlation with *Tp53* (r = 0.64) that reached a statistical tendency (*p* = 0.08). Moreover, lipotropes supplementation including vitamin B_6_ (LIP + Vt6) resulted in significant positive correlations between *Esr1* and *Tbx3* (r = 0.96; *p* ≤ 0.05) as well as *Hdac1* (r = 0.93; *p* ≤ 0.05).

## 4. Discussion

Breast cancer remains the most diagnosed cancer among women worldwide, with incidence continuing to rise despite advances in detection and treatment. The Developmental Origin of Health and Disease (DOHaD) framework posits that environmental exposures during critical developmental windows, particularly in utero, can have lasting effects on disease risk, partly through epigenetic programming such as DNA methylation. DNA methylation is the most widely reported epigenetic regulator of gene expression and a well-established hallmark of cancer. It represents a downstream outcome of one-carbon metabolism, a network of interconnected biochemical reactions that relies on the availability of dietary methyl donor nutrients, including folate, methionine, choline, and vitamin B_12_. Maternal intake of these nutrients during pregnancy is postulated to influence developmental programming and offspring disease risk in adulthood, including cancer susceptibility. Indeed, experimental studies indicate that maternal methyl donor nutrients supplementation can reduce mammary tumor risk in offspring; however, the molecular mechanisms underlying these protective effects remain unclear [10,13].

This study demonstrates that maternal exposure to methyl donor nutrients during pregnancy and lactation modulates mammary developmental gene signals in tumor tissues of adult female offspring, providing mechanistic insight into how early-life nutrition influences breast cancer susceptibility. Using a DMBA-induced estrogen receptor-positive mammary tumor model, we show that maternal lipotropes supplementation attenuates tumor-associated reactivation of fetal mammary developmental genes, suppresses epigenetic regulatory machinery, and disrupts coordinated estrogen receptor signaling central to mammary tumorigenesis.

### 4.1. DMBA-Induced Mammary Tumorigenesis Expresses Developmental and Epigenetic Dysregulation

Consistent with previous reports, DMBA exposure resulted in mammary tumors with ductal carcinoma in situ (DCIS) morphology, reflecting estrogen receptor-positive breast cancer [20,21,22]. At the molecular level, these tumors exhibited upregulation of genes involved in mammary development (*Tbx2*, *Tbx3*), epigenetic regulation (*Hdac1*, *Dnmt1*), and tumor progression (*Tp53*), consistent with evidence suggesting that mammary tumorigenesis involves partial reactivation of embryonic and developmental gene programs. Indeed, the T-box transcription factors TBX2 and TBX3 are critical regulators of embryonic mammary gland development and have been widely implicated in breast cancer progression [18,26]. TBX2 functions as a transcriptional repressor of tumor suppressor genes, thereby promoting proliferation and bypassing senescence checkpoints [27]. Interestingly, silencing Tbx2 has been shown to sensitize cisplatin-resistant breast cancer cells by reducing *p53* activation and shifting the cell cycle from S-phase arrest to a G2/M arrest, thereby enhancing treatment response [28]. Similarly, TBX3 directly represses *CDKN1A*, facilitating cell cycle progression and sustaining oncogenic growth signals [16,29].

Most studies in human breast cancer report increased Wnt10b expression, particularly in aggressive subtypes such as triple-negative breast cancer, where it is associated with activation of canonical Wnt/β-catenin signaling, enhanced proliferation, and stem-like phenotypes [30,31]. Activation of Wnt10b has also been reported in a DMBA-based model, but that study examined DMBA-induced cutaneous sebaceous neoplasms rather than mammary tumors [32], highlighting important tissue-specific differences in stem cell origin and tumor biology. *Wnt10b* expression in our study suggests that this pathway may not be a primary driver of tumorigenesis in our model, and that alternative Wnt-independent mechanisms may be involved. However, it is also important to note that the relatively small sample size may have limited the ability to detect subtle differences in *Wnt10b* expression.

Histone deacetylase 1 (HDAC1) is a key epigenetic regulator which modulates chromatin structure and gene expression by removing acetyl groups from histone proteins. Aberrant HDAC1 expression, most commonly overexpression, has been associated with enhanced breast cancer proliferation and migration [33,34]. DNMT1 is a critical epigenetic regulator responsible for maintaining DNA methylation patterns during DNA replication, thereby modulating gene expression and preserving genome stability. Aberrant overexpression of Dnmt1 is frequently observed in breast cancer, and consistent with our current findings, elevated levels have been reported in DMBA-induced mammary tumors [35,36].

CDKN1A, a well-characterized tumor suppressor, is frequently downregulated in both estrogen receptor-positive and triple-negative breast cancers; notably, loss of p21 has been shown to enhance the tumorigenic potential of DMBA [37,38]. p53 is a central tumor suppressor that is frequently mutated or functionally inactivated in human breast cancer. In contrast, in chemically induced models such as DMBA-driven mammary carcinogenesis, p53 is often retained in its wild-type form, and tumor development may occur despite the preservation of the gene sequence. In our study, p53 was significantly upregulated at the mRNA level in DMBA-induced tumors compared with normal mammary tissue, suggesting activation of a stress-responsive pathway in established tumors. In contrast, its downstream effector, *p21* was not affected.

### 4.2. Maternal Methyl Donor Nutrients Suppress Tumor-Associated Developmental Gene Reactivation

A central finding of this study is the reduction of *Tbx2* and *Tbx3* expression in mammary tumors from offspring exposed to maternal lipotropes supplementation. T-box transcription factors play critical roles in embryonic mammary gland development but are aberrantly re-expressed in breast cancer, where they promote proliferation, inhibit senescence, and enhance oncogenic signaling [14,15,16,17].

The attenuation of *Tbx2* and *Tbx3* expression following maternal methyl donor nutrients exposure suggests that early-life nutritional programming constrains the oncogenic reactivation of developmental regulators in adulthood. These findings support previous observations that maternal methyl donor nutrients supplementation reduces mammary tumor incidence by identifying specific developmental genes as downstream molecular targets [10,13]. Interestingly, our findings demonstrated no changes in *Wnt10b* mRNA expression in the lipotropes supplemented groups compared with both normal and DMBA-induced mammary tumors, perhaps suggesting context-dependent regulation and underscoring the distinction between transcriptional expression and functional pathway activation.

### 4.3. Maternal Micronutrient Status Modulates Epigenetic Regulatory Markers

Given the central role of one-carbon metabolism in providing methyl groups for DNA methylation, we evaluated key components of epigenetic regulation implicated in tumor progression, including *Mthfr* which regulates one-carbon metabolism, *Dnmt1* involved in DNA methylation, and *Hdac1* involved in conferring histone modification. Mammary tumors from cancer control offspring exhibited increased expression of *Hdac1* and *Dnmt1*, consistent with enhanced chromatin remodeling and DNA methylation activity during tumorigenesis [33,34,35,36,39]. Upregulation of *Mthfr* further suggests increased metabolic demand for methyl group generation in the tumor microenvironment.

Maternal lipotropes supplementation significantly reduced tumor expression of *Hdac1*, *Dnmt1*, and *Mthfr*, indicating that intrauterine methyl donor nutrients availability exerts long-term effects on epigenetic regulatory capacity in mammary tissues. These findings support the hypothesis that maternal nutrition influences breast cancer risk, at least in part, through persistent modulation of epigenetic machinery established during fetal development. Further, MTHFR polymorphisms have been associated with breast cancer susceptibility, with interaction with TP53 also reported [40]. While data are lacking on *Mthfr* activity in a DMBA-induced mammary tumor model, our findings are consistent with previous reports, with the diet-supplemented group revealing a significant decrease. Although methyl donor nutrients increase the availability of substrates required for DNA methylation, increased substrate supply does not necessarily result in upregulation of methylation enzymes. *Dnmt1* expression is largely regulated by proliferative and oncogenic signaling pathways rather than methyl donor nutrients abundance. Therefore, the observed reduction in *Mthfr*, *Dnmt1*, and *Hdac1* expression in the methyl donor nutrients-supplemented groups likely reflects attenuation of tumor-associated metabolic and epigenetic reprogramming rather than diminished methylation capacity, suggesting a normalization of the one-carbon metabolism–epigenetic regulatory axis during mammary tumorigenesis.

### 4.4. Modulation of Tumorigenesis and Cell Cycle Regulatory Genes

DMBA-induced tumors exhibited elevated expression of *Tp53* (*p53*), consistent with activation of DNA damage response pathways and cell-cycle checkpoint signaling triggered by carcinogen-induced genomic stress [41,42]. Such upregulation likely reflects a compensatory response to oncogenic insult rather than effective tumor suppression, as persistent DNA damage can lead to chronic activation of the p53-p21 axis without preventing malignant progression [43].

In contrast, tumors from offspring of dams fed the treatment diets showed reduced transcript levels of *Tp53*. This attenuation may indicate diminished oncogenic stress signaling or altered developmental programming of cell-cycle regulatory networks resulting from maternal nutritional exposure. Indeed, maternal diet has been shown to influence offspring mammary tumor susceptibility and associated molecular pathways long after early-life exposures [10,13,44,45].

Collectively, these findings suggest that maternal dietary intervention may modulate transcriptional activation of p53 during tumor development. The normalized expression of *Tp53* in tumors from lipotropes-exposed offspring may therefore reflect reduced proliferative or genotoxic stress, consistent with partial restoration of growth-regulatory signaling rather than direct enhancement of classical tumor suppressor activity. Notably, *Cdkn1a* expression was not significantly altered in our study; however, this apparent lack of effect may be attributable to the limited sample size and consequent reduced statistical power to detect more subtle transcriptional changes. However, it should be noted that although p21 and Tp53 are traditionally associated with tumor suppressive functions, their roles in cancer are highly context-dependent. Indeed, p21 can promote cell cycle arrest but has also been implicated in tumor survival and p53-independent resistance-conferring mechanisms [46]. Further, post-translational modifications of p53 can increase its stability while promoting cytoplasmic localization and reducing transcriptional activity, indicating that elevated p53 levels do not necessarily correspond to functional tumor suppressor activity [47].

### 4.5. Disruption of Estrogen Receptor–Developmental Gene Coupling

Maternal methyl donor nutrients availability reshaped *Esr1*-centered transcriptional networks in a manner that depended on tumor status and vitamin B_6_ inclusion. In non-cancer controls, *Esr1* and *Tbx3* were both downregulated while significantly positively correlated, suggesting a coordinated suppression of estrogen-driven developmental signaling under physiological conditions. In contrast, in cancer controls, both genes were upregulated, while *Esr1* was significantly positively associated with *Tbx2*, indicating synchronized activation of estrogen-responsive developmental pathways during tumorigenesis. Under full lipotropes supplementation (LIP + Vt6), *Esr1* expression was reduced and strongly positively correlated with *Tbx3* and *Hdac1*, while *Hdac1* was likewise downregulated, reflecting coordinated repression of estrogen signaling and chromatin remodeling.

Our findings advance current understanding by integrating developmental genes, epigenetic regulatory machinery, and estrogen signaling within a single mechanistic framework, an approach that has not been previously explored in the context of maternal methyl donor nutrition and mammary tumorigenesis. While these results offer a mechanistic insight, they are derived from a DMBA-induced rodent model and should therefore be interpreted within a preclinical context. Nonetheless, mechanistic studies are essential in this setting, as they help identify molecular targets and biomarkers that may enable risk stratification, inform early-life nutritional interventions, and guide prevention strategies aimed at modulating cancer susceptibility across the life course. However, our findings remain associative and do not establish causality. Further studies incorporating functional validation and clinical datasets are needed to determine the translational relevance of these mechanisms. Although clinical data were not collected, as this study was designed as a mechanistic extension of prior work [10,13], this represents both a limitation and a strength, providing a foundation for future investigations into genomic networks linking developmental programming to tumor outcomes, particularly focusing on developmental genes with oncogenic potential.

## 5. Conclusions

This study demonstrates that availability of maternal methyl donor nutrients during early development exerts lasting effects on mammary gene regulation in adult offspring. Within the paradigm of fetal programming, our findings indicate that maternal nutrition may shape the epigenetic landscape of the developing mammary gland, constraining the reactivation of fetal developmental pathways and reorganizing estrogen-centered transcriptional networks that influence tumor susceptibility later in life.

Notably, the lipotropes plus vitamin B_6_ diet reshaped the coupling pattern of *Esr1* with key regulatory partners, strengthening its coordinated association with *Hdac1* while modulating its relationship with *Tbx3*. Given the central role of ESR1 in mammary epithelial proliferation and estrogen responsiveness, this altered transcriptional coupling suggests epigenetically mediated reprogramming of estrogen-driven signaling rather than simple changes in individual gene expression levels. The data therefore support a model in which maternal methyl donor nutrients exposure reorganizes the regulatory architecture governing chromatin remodeling and developmental transcription factor activity, potentially limiting aberrant developmental reactivation and oncogenic estrogen signaling in adulthood. These integrated molecular changes provide an explanation for the reduced mammary tumor susceptibility previously observed following maternal lipotropes supplementation [10,13]. More broadly, this work expands the Developmental Origins of Health and Disease paradigm to breast cancer, identifying maternal nutrition as a determinant of long-term cancer risk.

Several limitations should be acknowledged. First, due to technical challenges with frozen tissue samples, we were unable to validate the transcriptional changes observed at protein level. Consequently, the functional implications of altered mRNA expression and gene–gene coupling remain to be confirmed. Indeed, while qRT-PCR provides sensitive quantification of transcriptional changes, mRNA levels do not always correlate with protein abundance due to regulation at translational and post-translational levels, including protein stability and degradation pathways. Previous studies have demonstrated that discrepancies between mRNA and protein expression are common in cancer biology and may reflect complex regulatory networks rather than direct functional outcomes [48]. Second, the relatively high dose of 7,12-dimethylbenz[a]anthracene (DMBA) used in this model likely induced rapid and aggressive tumor development, which limited our ability to distinguish subtle phenotypic differences between experimental groups and to evaluate tumor progression dynamics in detail. Comprehensive tumor characterization parameters, including incidence, latency, multiplicity, and growth progression, were not systematically captured, as the experimental design was primarily focused on mechanistic interrogation of tumor-associated gene expression. Consequently, the translation of molecular reprogramming into functional tumor behavior remains inferential. In addition, while qRT-PCR enabled sensitive quantification of transcriptional changes, mRNA expression levels do not necessarily reflect protein abundance or activity because of translational and post-translational regulatory processes. Furthermore, epigenetic modifications, such as DNA methylation and histone acetylation, were not directly assessed, and therefore mechanistic conclusions regarding epigenetic regulation remain theoretical. Nevertheless, these limitations do not diminish the value of this controlled model in enabling focused investigation of tumor-specific transcriptional reprogramming associated with maternal nutritional exposures. Future studies using optimized lower-dose DMBA models, integrated tumor phenotyping, and multi-level molecular validation will be important to more fully define the relationship between early-life nutritional exposures and tumor outcomes.

Future studies will address these limitations by optimizing carcinogen dosing, incorporating protein-level and functional assays, and directly interrogating epigenetic modifications to confirm the biological relevance of the transcriptional networks identified here.

## Figures and Tables

**Table 1:** Composition of the control (AIN 93G) and lipotrope-supplemented experimental diets.

Ingredients	Control	Lipotropes Plus Vitamin B_6_	Lipotropes Minus Vitamin B_6_
Casein	200	200	200
L-Cystine	3	3	3
Corn starch	397.486	393.486	393.486
Maltodextrin	132	132	132
Sucrose	100	100	100
Cellulose	50	50	50
Soybean oil	70	70	70
t-Butylhydroquinone (TBHQ), mg	0.014	0.014	0.014
Mineral mix	35	35	35
Vitamin mix	10	10	10
Choline bitartrate (41% Choline)	2.5	12.5	12.5
Choline, g/kg	1.0	5.1	5.1
Cyanocobalamin (B12), μg/kg	25	125	125
Folic acid, mg/kg	2.1	10.0	10.0
Methionine, g/kg	5.1	9.0	9.0
Pyridoxine HCl (B6), mg/kg	7.0	35.0	7.0
Caloric density (kcal/g)	4.00	4.00	4.00

**Table 2:** Pearson’s correlation analysis of estrogen receptor 1 (*Esr1*) with genes involved in mammary developmental process, epigenetic modifications, and cancer biology.

Pearson Correlation Coefficient								
Genes [r (*p*-Value)]								
Groups	*Tbx2*	*Tbx3*	*Wnt10b*	*Cdkn1a*	*Tp53*	*Dnmt1*	*Hdac1*	*Mthfr*
Non-cancer control	0.68 0.09 ^#^	0.89 0.007 *	0.27 0.56	0.05 0.91	0.11 0.81	0.23 0.62	0.42 0.35	0.65 0.11
Cancer control	0.74 0.04 *	0.59 0.13	0.40 0.32	0.24 0.57	−0.20 0.64	0.08 0.84	0.36 0.38	0.30 0.47
LIP − Vt6	−0.20 0.64	0.18 0.67	0.09 0.84	−0.56 0.15	0.64 0.08 ^#^	−0.49 0.22	−0.02 0.96	0.09 0.84
LIP + Vt6	0.58 0.17	0.96 0.001 *	−0.22 0.64	−0.18 0.69	0.69 0.09	0.06 0.69	0.93 0.003 *	0.45 0.31

*Tbx2*: T-box transcription factor 2; *Tbx3*: T-box transcription factor 3; *Wnt10b*: wingless-type MMTV integration site family, member 10B; Cdkn1a: cyclin-dependent kinase inhibitor 1A (p21); *Tp53*: tumor protein 53; *Dnmt1*: DNA methyltransferase 1; *Hdac1*: histone deacetylase 1; *Mthfr*: methylenetetrahydrofolate reductase; LIP − Vt6: lipotropes supplementation excluding vitamin B_6_; LIP + Vt6: lipotropes supplementation including vitamin B_6_; * statistically significantly different; ^#^ *p* < 0.1, indicating a trend.

## Data Availability

Dataset available on request from the authors.

## Abbreviations

The following abbreviations are used in this manuscript:

Cdkn1a	Cyclin-Dependent Kinase Inhibitor 1A
DMBA	7,12-Dimethylbenz[a]anthracene
Dnmt1	DNA (Cytosine-5)-Methyltransferase 1
DOHaD	Developmental Origins of Health and Disease
Esr1	Estrogen Receptor 1 (gene symbol, rat)
HDAC1	Histone Deacetylase 1
Hprt1	Hypoxanthine Phosphoribosyltransferase 1
LIP+VB6	Lipotropes-Supplemented Diet Including Vitamin B6
LIP-VB6	Lipotropes-Supplemented Diet Excluding Vitamin B6
Mthfr	Methylenetetrahydrofolate Reductase
qRT-PCR	Quantitative Real-Time Polymerase Chain Reaction
SEM	Standard Error of the Mean
Tbx2	T-Box Transcription Factor 2
Tbx3	T-Box Transcription Factor 3
Tp53	Tumor Protein p53
Wnt10b	Wingless-Type MMTV Integration Site Family, Member 10B
